# Fruits and barks extracts of *Zanthozyllum heitzii* a spice from Cameroon induce mitochondrial dependent apoptosis and Go/G1 phase arrest in human leukemia HL-60 cells

**DOI:** 10.1186/0717-6287-47-54

**Published:** 2014-10-23

**Authors:** Constant Anatole Pieme, Guru Kumar Santosh, Emmanuel Mouafo Tekwu, Tülin Askun, Hatice Aydeniz, Jeanne Yonkeu Ngogang, Shashi Bhushan, Ajit Kumar Saxena

**Affiliations:** Department of Biochemistry and Physiological Sciences, Faculty of Medicine and Biomedical Sciences, University of Yaoundé 1, PO BOX 1364, Yaoundé, Cameroon; Cancer Pharmacology Division, Indian Institute of Integrative Medicine, Canal Road, 18001 Jammu, India; Laboratory for TB research, Biotechnology Centre, Faculty of Science, University of Yaoundé 1, PO BOX 812, Yaoundé, Cameroon; Department of Biology, Faculty of Sciences and Arts, University of Balikesir, Cagis Campus, 10145 Balikesir, Turkey

**Keywords:** *Zanthozyllum heitzii*, Syringic acid, Apoptosis, HL-60 cells, ROS, Mitochondrial membrane potential

## Abstract

**Background:**

*Zanthoxylum heitzii* is a spice used to prepare several dishes and to treat tumors, syphilis, malaria, cardiac palpitations, urogenital infections in the west region of Cameroon, but the antitumor mechanisms and chemical composition are not yet investigated.

This study was aimed to determine the antiproliferative effects of four extracts from the fruits and barks of *Zanthoxyllum heitzii* (Rutaceae) on apoptosis in human promyelocytic cells, their mechanisms and the chemical composition. The 3-(4, 5-dimethylthiazole-2-yl)-2,5-diphenyltetrazolium bromide (MTT) assay was used to determine the fifty percent inhibition (IC_50_) concentration of the cell lines after treatment. The effect on morphology was observed using a light or fluorescence microscopy. The rate of apoptosis and the cell cycle were measured using flow cytometry (FCM). The phytochemical analysis of the extract was carried with HPLC/MS methods.

**Results:**

The phytochemical analysis of the extracts indicated the presence of four known polyphenols (Syringic acid, Juglon, Luteolin and Myricetin) in both fruits and barks of *Z. heitzii* but in different quantities. Syringic acid and Myricetin concentrations were between 17-21 fold higher in the fruits than the stem bark. Rhamnetin (393.35 μg/mL) and Oleuropein (63.10 μg/mL) were identified only in the stem barks of *Z. heitzii*. Among the four extracts tested for cytotoxicity properties, only the methanol extract of fruits and barks significantly inhibited cell proliferation of HL-60 cells with IC_50_ value of 20 μg/mL and 12 μg/mL respectively. HL-60 cells treated with *Z. heitzii* extracts significantly produced reactive oxygen species (ROS) with concurrent loss of mitochondrial membrane potential (MMP). Modifications in the DNA distribution and enhanced of G1/G0 phase cell cycle arrest were observed in a concentration dependent manner.

**Conclusions:**

Polyphenols from *Z. heitzii* plant exert inhibitory effect on HL-60 cells through the reactive oxygen species (ROS) generation, loss of mitochondrial membrane potential and cell cycle destabilization.

## Background

Phenols are compounds possessing one or more aromatic rings with one or more hydroxyl groups. They are broadly distributed in the plant kingdom and are the most abundant secondary metabolites of plants, with more than 8,000 phenolic structures currently known, ranging from simple molecules (phenolic acids) to highly polymerized substances (tannins). The beneficial effects of dietary polyphenols on human health have been widely assumed to act through various biological effects such as free radical scavenging, metal chelation, modulation of enzymatic activity and altering signal transduction pathways [1, 2]. Phytochemical research has shown that tea contains a large number of polyphenols with different chemical structures (amino acids, catechins, purine alkaloids, and chlorogenic acid) each imparting unique biological properties [2, 3].

*Zanthoxylum heitzii* (Aubrev. & Pellegr) or *Fagara heitzii* is a plant from Rutaceae family [4]. Plants from this family are distributed in 150 genera and 1500 species. They are commonly found in tropical and warm temperate regions in the world [4, 5]. Some are used in the manufacturing perfumes and/or in the food industry as well as in traditional medicine. Previous investigations have reported that the phytochemical composition of *Z. heitzii* include amides, lignanes [6], alkaloids such as benzophenanthridines (nitidine, methylnitidine etc.) steroids and terpenes [7, 8]. Enormous molecules have been isolated from the stem bark of *Z. heitzii* such as heitziamide A, heitziamide B, heitziethanoid A, heitziethanoid B, trans-fagaramide, arnottianamide, iso-c fagarine, iso-skimmianine, arctigenin methyl ether, savinin, (+)-eudesmin, (+)-sesamin, lupeol, lupeone, β-sitosterol, stigmasterol and stigmasterol-3-O-β-D-glucopyranoside [6]. *Z. heitzii* is widely used in central Africa for the treatment of many diseases such as cancers, syphilis, malaria, cardiac palpitations and urogenital infections [6, 9]. Dry powder of fruits of *Z. heitzii* is used as a spice for the preparation of “Nkui” and “Nah poh”, two dishes in Cameroon [10]. The bark extracts of *Z. heitzii* is used as an insecticide and against cardiac affections [11]. Other biological properties of the aqueous extract of the fruit of *Z. heitzii* its bark and stem have been previously investigated [12, 13]. Fagaricine, an aqueous extract formulation from the root of *Z. heitzii* was used as an immune-restorative phytomedicine to treat immunodeficiencies [14].

However, no study has been reported on the cytotoxicity properties and the mechanism of inducing apoptosis by the barks and fruits extracts of *Z. heitzii* on human promyelocytic leukemia HL-60 cells. Therefore this study reports the cytotoxic and apoptotic activities of *Z. heitzii* root and fruits extracts on HL-60 cells.

## Results and discussion

### Results

#### Phytochemical analysis of the extracts

**Figure 1:**
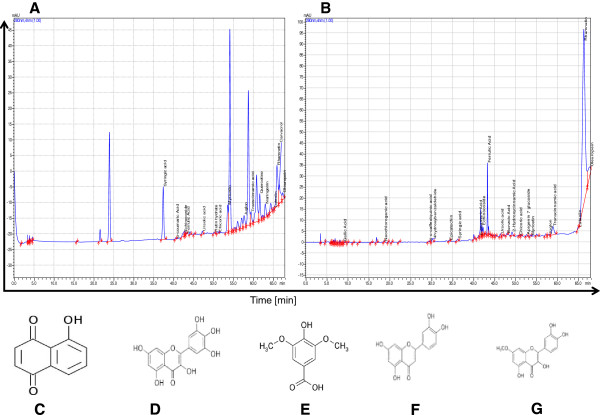
**Profile of the polyphenols compound present in** ***Z. heitzii*** **extracts.** Data are obtained after analysis with a LC-MS analysis following by identification with several phenolic compounds as standards. **(A)** Fruits; **(B)** barks; **(C)** Juglon; **(D)** Myricetin; **(E)** Syringic acid; **(F)** Luteolin; **(G)** Rhamnetin.

For further interpretation of the observed effects of *Z. heitzii* extracts, it is important to know the main molecules present in the extracts. For this purpose, we carried out a LC-MS analysis and identified several phenolic compounds using HLPC-LC/Ms methods. The chromatographic profiles of the extracts from fruit and the barks of *Z. heitzii* are shown in Figure 1A and 1B. The results showed that molecules found in the barks and fruits are quantitatively and qualitatively different (Table 1). Twelve different molecules were identified with the concentrations varying from 3.68 to 1087.29 μg/mL. Eight major polyphenols compounds (>15 μg/mL) were identified in the fruits and barks of *Z. heitzii* extracts respectively. Four molecules including Syringic acid, Myricetin, Juglon and Luteolin were found in both extracts (Table 1). The concentration of these molecules was between 1– 21 fold higher in the fruits compared to barks of *Z. heitzii* extracts. Rhamnetin and Oleuropein present in the barks extract were absent in the fruits. These polyphenolic compounds have been involved in the cytotoxicity or G0/G1 phase arrest in different cancer cell lines.

**Table 1:** **Characteristics of molecules from** ***Z. heitzii*** **extracts**

Phenolic groups	Molecules from extract	***Concentration (μ/mL)***	
		Barks	Fruits
Phenolic acids	Chicoric acid	*	9.4355
	Neochlorogenic acid	7.26536	*
	4 - 0 caffeolquinic acid	17.31354	*
	Syringic acid	61.61854	1087.2937
Flavonoids	Apigenin 7 glucoside	16.75124	*
	Luteolin	11.90008	14.8787
	Myricetin	23.03052	505.77894
	Naringenin	*	26.94684
	Rhamnetin	393.35814	*
Other polyphenols	Carvacrol	*	8.1243
	Juglon	11.4294	59.11246
	Oleuropein	63.10464	*

(*Concentration lower than 5 μg/ml).

#### Cytotoxicity effect of *Z. heitzii*extracts on HL-60 cells

**Figure 2:**
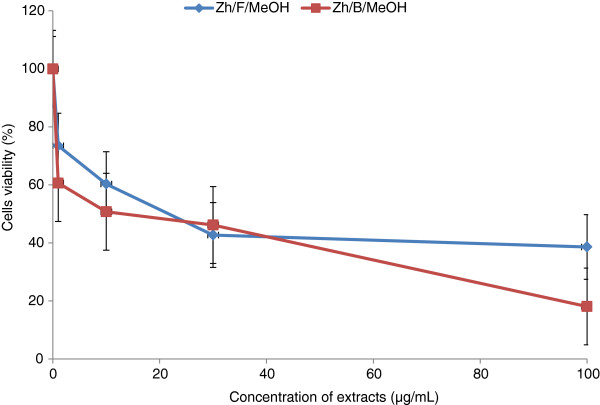
**Inhibitory effects and cytotoxicities of** ***Z. heitzii*** **extracts.** HL-60 cells were treated with different concentrations of Z*. heitzii* for 48 h in triplicate. Cell viability was determined by MTT assay and indicated in percentage of cell viability of three independent assays.

To evaluate the anti-proliferation property, HL-60 cells lines were treated with *Z. heitzii* extracts at 1, 10, 30 and 100 μg/mL and cell viability was detected by the MTT method. As shown in Figure 2, only methanol extracts of *Z. heitzii* significantly inhibited the growth of HL-60 cell lines after 48 h. The IC_50_ was 20 and 12 μg/mL respectively for the fruits and barks extracts (Table 2). The barks extract of *Z. heitzii* was most active than fruits on HL-60 cells. The very lower cytotoxicity effects of the methanol and aqueous extracts were found in human prostate cancer PC-3 cell lines (IC_50_ > 100 μg/mL) and these extracts cannot as lead candidate for anticancer activity according to National Cancer Institute (NCI).

**Table 2:** **Fifty percent cell growth inhibition (IC**_**50**_ **) of different extracts of** ***Z. heitzii***

Cell lines	IC _50_ (μ/mL)			
	Z _h_ /F/H _2_ O	Z _h_ /F/MeOH	Z _h_ /B/H _2_ O	Z _h_ /B/MeOH
HL-60	>100	20 ± 2.3	>100	12 ± 1.5

Results are expressed as mean ± SD; n = 3; Z_h_/F/H_2_O: Aqueous extract of fruits; Z_h_/B/H_2_O: aqueous extract of barks; Z_h_/F/MeOH: Methanol extract of fruits; Z_h_/B/MeOH: Methanol extract of barks.

#### Morphological changes of treated HL-60 cells with *Z. heitzii*extract

**Figure 3:**
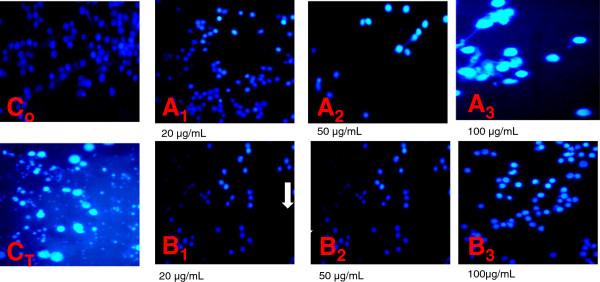
**Effect of** ***Z. heitzii*** **extract on nuclear morphological changes of HL-60 cells.** After 24 h of treatment cells were stained with Hoechst 33258 incubated for 30 min, and observed under fluorescence microscope. Olympus, Tokoy, Japan magnification X 200). (C_o_) Control; (C_T_) Camptotecin (2 μM); (A) Methanol extract of fruits *Z. heitztii* (A_1_: 20 μg/mL; A_2_: 50 μg/mL; A_3_:100 μg/mL; (B) Methanol extract of barks of *Z. heitztii* (B_1_: 20 μg/mL; B_2_: 50 μg/mL; B_3_:100 μg/mL).

The characteristic apoptotic morphological changes of HL-60 treated cells were assessed by the fluorescent microcopy after staining with Hoechst dye. Because of the integrity of the cell membrane, Hoechst dye was unable to infiltrate into the HL-60 cells when the cells were alive or still in the early process of apoptosis, while the dead cells have Hoechst inside and the nuclei were stained a bright red color. The results revealed nuclear condensation, membrane blebbing, nuclear fragmentation and apoptotic bodies (the hallmarks of apoptosis) in cells that had been incubated with *Z. heitzii* extracts (Figure 3). The control cells did not exhibit any of the above morphological changes, the nuclei were less stained in bright blue and the color was homogeneous (Figure 3C_o_). Chromatin condensation and other apoptotic characters were observed only in the treated cells. After treating with *Z. heitzii,* at 20 μg/mL for 24 h, the blue emission light in apoptotic HL-60 cells was much brighter than the control cells (Figure 3). When, the concentration of *Z. heitzii* increased, the numbers of cells emitting the blue light increased demonstrating apoptotic cells. Condensed chromatin was also found in many *Z. heitzii*-treated cells with the apoptotic bodies at 100 μg/mL (Figure 3A_3_). This result suggests that at this concentration, the fruits extract of *Z. heitzii* can induce apoptosis better than the barks extract (Figure 3A_3_ and 3B_3_).

#### Reactive oxygen species (ROS) production by HL-60 cells treated with *Z. heitzii*extract

**Figure 4:**
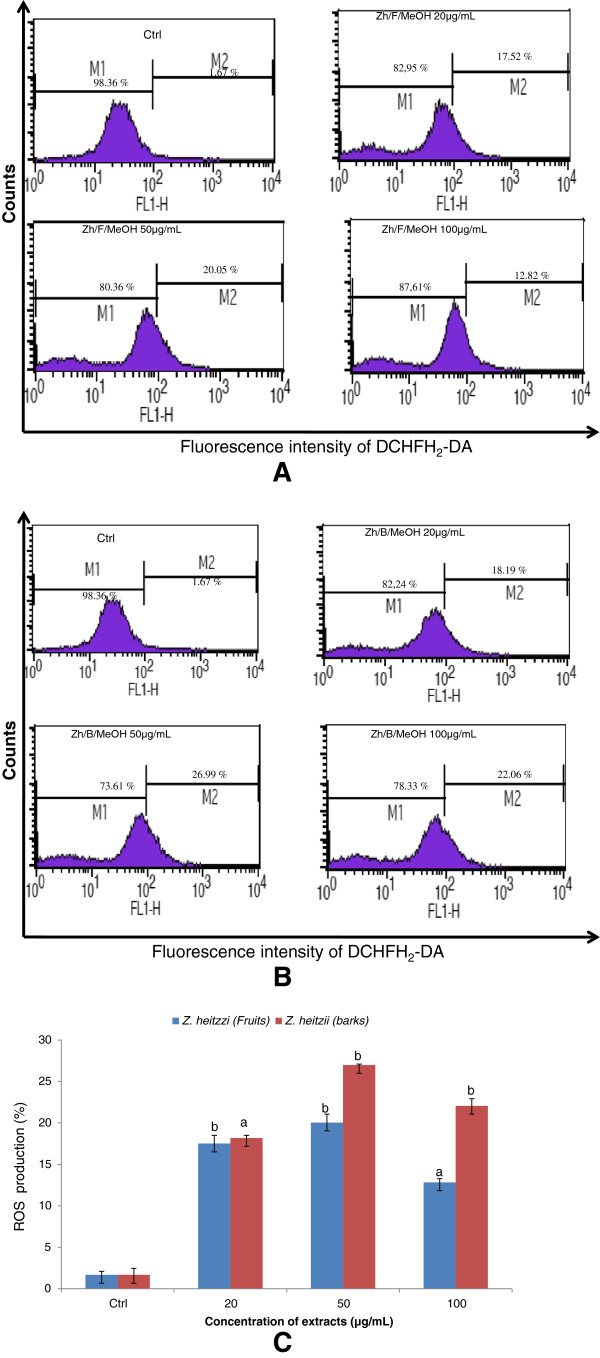
**Effects of** ***Z. heitzii*** **extract on ROS production in HL-60 cells.** Cells were treated with extract for 24 h followed by staining with DCHFH_2_-DA (5 μM), incubated for 30 min and the fluorescence in the cells was immediately analyzed using flow cytometry. Data presented are the fluorescence intensity at different concentrations; Control; 20 μg/mL; 50 μg/mL; 100 μg/L. **(4A)** MeOH fruits extract; **(4B)** MeOH barks extract; **(4C)** is a bold graphic variation of ROS production by HL-60 cells treated with the two extracts of *Z. heitzii* for 24 h. The fluorescence in the cells (staining with DCHFH_2_-DA) is represented as the percentage of ROS production considers rewording immediately analyzed using flow cytometry. Data shown are expressed as mean ± SD Values with different letter are significantly different (p < 0.05).

Recently, studies indicated that ROS played an important role in the induction of apoptosis. To examine whether ROS production is an essential event for *Z. heitzii* -induced apoptosis, we determined the generation of ROS by human promyelotic leukemia cells after 24 h of treatment. An increase in ROS production was observed when higher concentration both fruits and barks extracts of *Z. heitzii* were used (Figure 4A and 4B). In comparison to the control, an increased ROS production ranging between 7.67 to 12.00% for fruits and 10.89 to 16.16% for barks extracts of Z*. heitzii* was found (Figure 4C). The results show that both fruits and barks extracts induce ROS production in HL-60-treated cells. However barks extract of *Z. heitzii* induces greater ROS production than fruits extract (Figure 4C).

#### Effect of *Z. heitzii*extracts on the mitochondrial membrane potential of HL-60 cells

**Figure 5:**
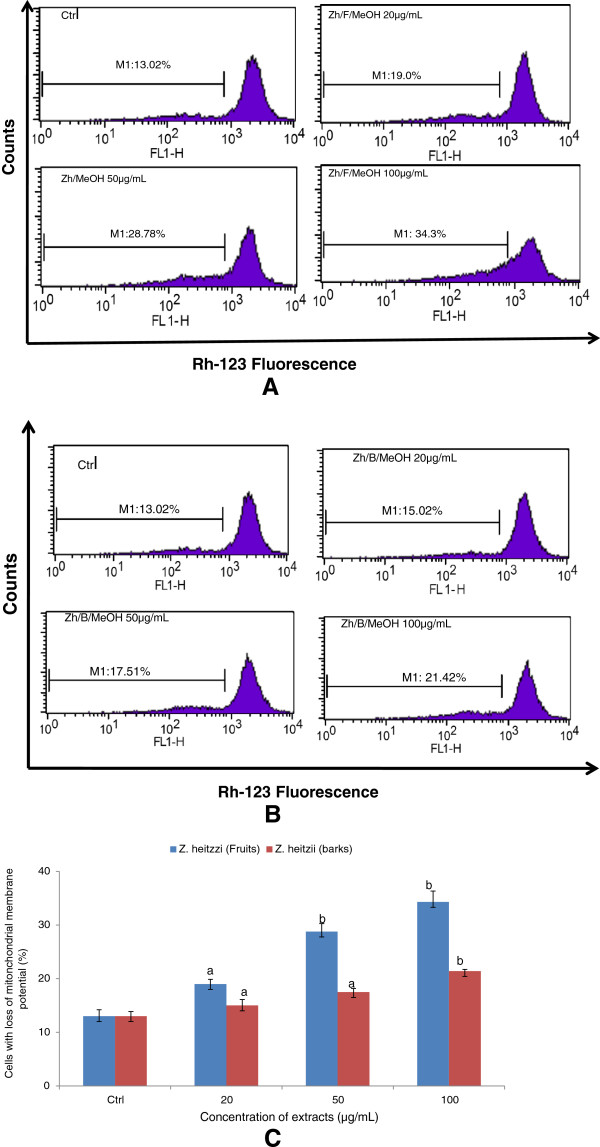
**Effects of** ***Z. heitzii*** **extracts on the integrity of mitochondrial membrane.** HL-60 cells were treated with extract and incubated 1 h with 200 nM of Rh-123 and then analyzed by flow cytometry. Data presented are the fluorescence intensity at different concentrations of extract; Control; 20 μg/mL; 50 μg/mL; 100 μg/mL. **(5A)** MeOH fruit extract; **(5B)** MeOH bark extract. **(5C)** graphic representation of mitochondrial damage of HL-60 cells treated with the two extracts of *Z. heitzii* for 24 h. The fluorescence in the cells is represented as the percentage the mitochondrial damage immediately analyzed using flow cytometry. Data shown are expressed as mean ± SD, Values with different letter are significantly different (p < 0.05).

Reactive oxygen species once generated cause massive oxidation of redox sensitive proteins and lipids leading to mitochondrial damage. Since the alteration of the mitochondrial function plays a major role in the apoptotic process, we investigated the effects of the extracts of *Z. heitzii* on the mitochondrial potential of HL-60 treated cells. This was examined by measuring their ability to retain Rhodamine 123, a fluorescent dye used to indicate the loss of mitochondrial transmembrane potential. Under this condition, the control HL-60 cells showed 13.05% loss fluorescence. After treatment with either fruits or barks extracts of *Z. heitzii,* the loss of the fluorescence increased with the concentration of extract demonstrating that the mitochondrial membrane potential dysfunction varied with the mitochondrial membrane potential (Figure 5A and 5B). A linear increase (r^2^ = 0.988; r^2^ = 0.975) of mitochondrial membrane potential disruption was found either with fruits and barks extracts of *Z. heitzii* respectively (Figure 5C). However, the fruits extract of *Z. heizii* exhibited higher mitochondrial potential dysfunction than barks extract of the same plant. These findings support that extracts of *Z. heitzii* altered the mitochondrial membrane potential.

#### Effect of *Z. heitzii*extracts on DNA content and cell cycle of HL-60 cells

**Figure 6:**
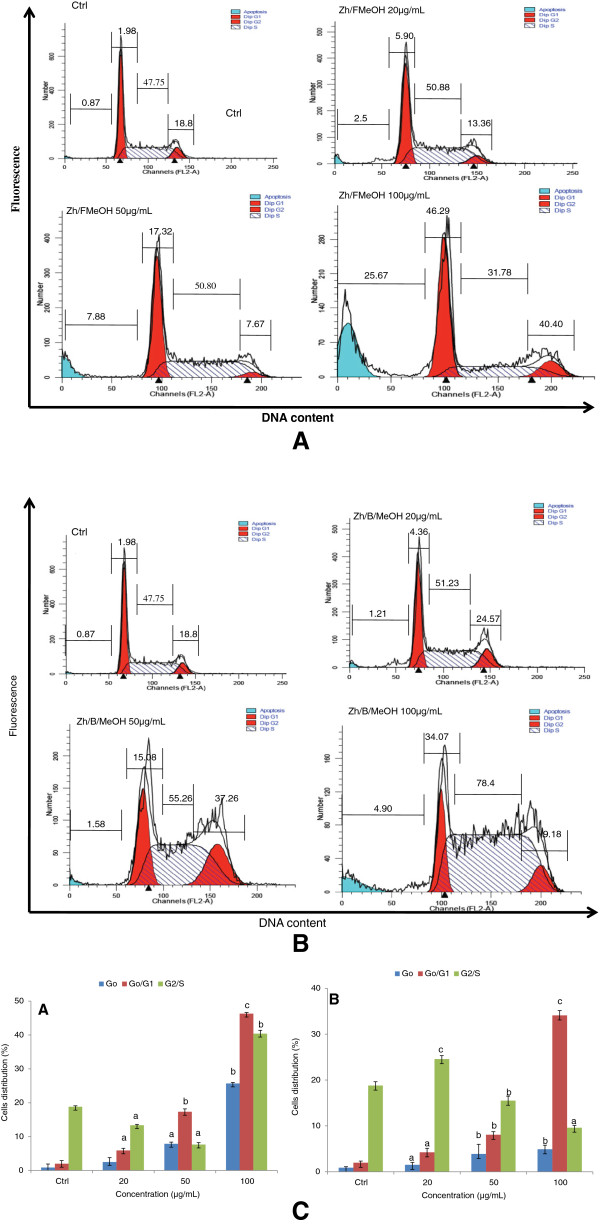
**Cell cycle analysis of** ***Z. heitzii*** **extracts in HL-60 cells after 24 h.** Treated cells were incubated with RNAse (40 μg/mL) stained with propidium iodide (25 μg/mL) and analyzed by BD-FACS Caliber flow-cytometer; **(6A)** MeOH fruit extract; **(6B)** MeOH bark extract; Ctrl: Control; 20 μg/ml; 50 μg/mL; 100 μg/mL; **(6C)** Cell cycle phase (G0/G1, M, G2/M) of the fruit (A) and bark (B) extracts of *Z. heitzii* (barks). Data shown are expressed as mean ± SD, Values with different letter are significantly different in the group (p < 0.05).

To investigate the mechanisms by which *Z. heitzii* induced cytotoxic effects in HL-60 cells, we cultured the cells for 24 h in the presence of Z*. heitzii* extracts, the DNA content and the cell cycle were analyzed by flow cytometry. The results presented in Figure 6A and 6B demonstrated that the extracts of *Z. heitzii* induced apoptosis in HL-60 promyelocytic cells in different manners. The cell cycle profile was not modified when the cells were treated with both extracts at the lowest concentration. The same observation was noted when the cells were treated with the fruits extract of *Z. heitzii* at 100 μg/mL*.* An exponential increase of the G0 (r^2^ = 0.99) and G0/G1 (r^2^ = 0.99) phases was observed respectively from 2.5 to 25.68% and 5.91 to 46.29% for the fruits extract of *Z. heitzii* after treatment of cells with different concentration of extract (Figure 6A). The barks extract of *Z. heitzii* exhibited an increase linear (r^2^ = 0.953) G0 phase (from 1.58 to 7.90%). This was accompanied by the decrease of G2/M phase (from 24.57 to 9.62) (Figure 6B). In addition, the G0/G1 nuclei population of HL-60 cells increased in a concentration-dependent manner with the extract. However, both fruits and barks extracts of *Z. heitzii* induced a prominent G0/G1 population arrest in HL-60 cells at 100 μg/mL (Figure 6A and 6B). This result confirmed that *Z. heitzii* extracts induced apoptosis through the G0/G1 arrest phase.

### Discussion

It has been reported that extracts from natural products, such as fruits, vegetables and medicinal herbs, have positive effects against cancers, compared with chemotherapy or recent hormonal treatments [15]. There is increasing evidence that many natural isolated compounds and Cameroonian medicinal herbs are biological modifiers of cancer treatment [16]. The hypothesis of this study was to confirm that a crude extracts of *Z. heitzii* could impact cell viability of HL-60 cells via apoptosis. Our data demonstrated that crude extracts of Z*. heitzii* treatment of the HL-60 cells induced a significant inhibition of cells (cytotoxicity) (Figure 2 and Table 2) and apoptosis in a concentration-dependent manner. The extract has a potent inhibitor effects on cell viability and can induce apoptosis of HL-60 cells.

Apoptosis induced by crude extracts of Z*. heitzii* was confirmed by characteristic DNA ladders in the cell cycle analysis (Figure 6A and 6B) and cellular morphological changes (Figure 3). These findings are relevant because the regulation of apoptotic machinery is important in the development of cancer disease. One of the main goals of anticancer potential of any drug/extract is the induction of apoptosis in cancer cells. Apoptosis or programmed cell death is one of the most important targets for cancer treatment comprising chemotherapy as well as chemoprevention. It is characterized by membrane blebbing, cytoplasmic condensation, formation of apoptotic bodies, DNA fragmentation, alteration in membrane symmetry, activation of cascade of caspases and loss of mitochondrial membrane potential [17].

Extracts of *Z. heitzii* cause a synergetic induction of apoptosis and mitochondrial damage in human leukemia HL-60 cells. Nowadays mitochondria have been proposed as a novel prospective target for chemotherapy-induced apoptosis. Several chemotherapy agents can cause apoptosis by destroying to the mitochondria. Partial disruption of mitochondrial membrane potential occurs early in apoptosis, this reduction of the mitochondrial membrane potential (MMP) may be due to an opening of mitochondrial permeability transition pores [18]. We noted a significant decrease of MMP in the cells treated with extracts of *Z heitzii* (Figure 5) suggesting that the extracts induce changes of the mitochondrial function. In many cells, morphological and molecular changes in mitochondria are a crucial stage in apoptosis induced by *Z. heitzii* extracts [19]*.* Mitochondria are a source of ROS during apoptosis and the reduction of mitochondria membrane potential leads to increased generation of ROS and apoptosis [20]. ROS are generated in and around mitochondria are regarded as the byproducts of normal cellular oxidative processes. Many anti-cancer drugs act as pro-oxidants targeting mitochondria which are involved in the generation of free radicals such as reactive oxygen/nitrogen species eventually leading to the activation of apoptosis [21, 22]. Our results showed an increase in the ROS production in the cells treated with both fruits and barks extracts of *Z. heitzii* with the maximum noted at 50 μg/mL (Figure 4). However, at 100 μg/mL the reduction of ROS production was noted while the mitochondria damage increased. This finding demonstrated that *Z. heitzii* extracts have limited effects on ROS regeneration and might have also antioxidant activity. Therefore, our data suggests the possibility that *Z. heitzii* might penetrate into cells and directly target mitochondria to increase membrane permeability and decrease membrane mitochondrial potential (Δψm) accompanied by ROS production at lower concentration. Several studies have reported that ROS act as secondary messengers in apoptosis induced by anti-cancer and chemopreventive agents [23, 24]. ROS, such as superoxide anion and hydrogen peroxide, are toxic byproducts of cellular metabolism. Although ROS at moderate concentrations are not toxic but rather act as signaling molecules, their overproduction and/or accumulation can cause non-specific damage to proteins, other cellular components and nuclear acids [25, 26]. The perturbation of cell-cycle progression by alteration of DNA content plays a vital role in the proliferation of cancer cells. Cell cycle arrest is one of the main targets of many anticancer drugs such as camptothecin, doxorubicin, cisplatin, 5-fluorouracil. It has been shown that the ability of molecules/drugs to arrest cell cycle in G2/M or S phase was related to their sensitivity [27]. Here arises a question whether G2/M or G0/G1 phases arrest induced by *Z. heitzii* extracts is the predominant pathway for cytotoxic effects in HL-60 cells or not. Our results showed an accumulation of G0/G1 phase when the HL-60 cells were treated with both fruits and barks extracts of *Z. heitzii*. The proportion of cells with DNA content in G0 phase also grew continuously. The induction of cell cycle arrest is not a separate event; rather the cell cycle arrest leads to apoptotic cell death.

This study demonstrated that the modification of cell cycle profile was found at concentration higher than 20 μg/mL mainly with the fruit extracts of *Z. heitzii.* This finding suggests that apoptosis or G0 phase arrest induced by *Z. heitzii* extract occurs through different mechanisms in the two parts of plant: the accumulation of G0/G1 phase with both fruit and bark extracts and the reduction of G2/M phase only observed in cells treated with bark extracts (Figure 6A and 6B). Our results demonstrated that significant apoptotic effects and G0/G1 phase arrest occurred in HL-60 cells treated at 100 μg/mL. In this study, apoptotic cell death is preceded by an arrest of cell cycle and an accumulation of cells in the G2/M-phase at the expense of the G0/G1-phase. We found that *Z. heitzii* extract induces ROS generation-dependent cell cycle arrest at the G0/G1 phase, followed by a late apoptosis in HL-60 cells. Generally, apoptosis is initiated by either an extrinsic (activated caspase-8) or an intrinsic pathway (activated caspase-9) [28]. The extrinsic pathway can directly activate caspase-8 through death receptors on the cell surface. The intrinsic pathway regulates apoptotic cascades by the signaling convergence in the mitochondrion, which results in the alteration of the membrane mitochondrial potential (MMP), the release of cytochrome C into the cytosol, and the activation of caspase-9 [29]. Since our results showed a damage of mitochondria membrane, we presumed the mitochondrial-mediated cell death pathway was being activated by extracts of *Z. heitzii.*

Identification of the phytochemical compounds from plant extracts responsible for apoptosis may have important implications in cancer therapy. Several studies demonstrated the beneficial effects of plant phytochemical compounds such as tannins, alkaloids, polyphenols and flavonoids from different plants from Rutacea family. Plants from this family are known to contain appreciable amount of polyphenols which have many health promoting benefits. Our study revealed the presence of three groups of polyphenols (flavonoids, phenolic acids and others). Several studies have shown that these phytochemical molecules to have health benefits [30–33]. Syringic acid, Luteolin, Myricetin and Juglon identified in *Z. heitzii* extracts have been reported to have antiproliferative, anticancer, cytotoxicity property [34–39], antioxidant [40–42] and other biological properties. The antiproliferative and apoptosis properties of *Z. heitzii* demonstrated in this study could be attributed either to the presence of individual or synergetic activity of the main molecules Syringic acid, Luteolin, Myricetin and Juglon or to the combination of these molecules with other unknown compounds that have not been identified by HPLC-MS method. Difference between the apoptotic effects of barks and fruits extracts of *Z. heitzii* is probably due either to variation found in quality and quantity of molecules found in each extract or due to the antagonistic effects of these molecules which can reduce their antiproliferative activity. However, the mechanism by which *Z. heitzii* induces G0/G1 phase cell arrest and apoptosis is not totally elucidated, but a clue may reside in its ability to increase ROS production through intrinsic pathway that regulates apoptosis.

## Conclusion

Our datas indicate that *Z. heitzii* induced apoptosis in HL-60 cells via ROS generation and mitochondrial pathway. However the entire mechanism involved in apoptosis and the main molecule that mediates this process yet needs to be ascertained.

## Methods

### Plant material and extraction

Fruits and barks of *Zanthoxyllum heitzii* (Rutaceae) were collected on the 30^th^ June 2010 at Batchingou in the west of Cameroon and identified under the reference number 1441/HNC of the National Herbarium of Cameroon where the voucher specimen is deposited there. Air-dried fruits and barks of *Z. heitzii* were ground and an aliquot (150 g) of each powder was extracted separately by maceration (72 h) in 1.5 L of water and methanol. The same procedure was repeated once with the same residues. Each mixture was filtered and concentrated to dryness. Each crude extract was stored at 4°C and freeze - dried for further studies.

### Cell culture

Two cell lines Prostate cancer (PC-3) and Human promyelocytic leukemia (HL-60 cells) was obtained from European Collection of Cells Culture (ECCC), Sigma Aldrich, India and used for the antiproliferative screening. They were grown in RPMI-1640 medium containing 10% Foetal bovine serum (FBS), penicillin (100UI/mL) and streptomycin (100 μg/mL medium). The cells were cultured in the incubator (Thermocom Electron Corporation, USA) at 37°C, 5% CO_2_; 98% humidity. The cells were used for different assays during logarithmic growth phase while the untreated control cultures received only the vehicle (DMSO <0.1%).

### Cells viability and treatments

The human promyelocytic leukemia HL-60 cells were seeded in different 96 well plates containing 15×10^3^ and 6 ×10^3^ cells/100 μL/well, respectively. The cultured cells were treated by the addition of 100 μL of serial dilutions of the *Z. heitzii* extracts dissolved in DMSO to give a final concentration of 100, 30, 10 and 1 μg/mL. The process was done in triplicates. For prostate cancer cells (PC-3), the extract was added after 24 h of incubation. In addition, the DMSO alone was added to another set of cells as the solvent control (DMSO <0.1%). The cells were then incubated for another 48 h prior to the addition of 20 μL of 2.5 mg/mL solution of 3-(4, 5-dimethylthiazol-2-yl)-2, 5-diphenyltetrazolium bromide (MTT) into each well. The incubation was continued for another 3 h before the media was removed. A mixture of DMSO (150 μL) was added to each well and mixed to ensure dissolving of the crystal formazan before the absorbance at 570 nm was measured. Three replications of each experiment were performed and the fifty percent inhibitory concentration (IC_50_) of each extract was calculated. The extract and the cells which show IC_50_ lower than 20 μg/mL were used to continue the study.

### Hoechst 33258 staining of cells for nuclear morphology

HL-60 cells (2×10^6^ cells/3mL/well) were treated with *Z. heitzii* extracts at different concentration of extract for 24 h. They were collected and centrifuged at 400 g and washed once with PBS. A solution of Hoechst (Hoechst, 10 μg/mL; citric 10 mM; Na_2_HPO_4_ 0.45 M; Tween-20 0.05%) was added in each tube and kept in the dark at room temperature for 30 min. The mixture was then washed once with PBS and the pellet resuspended in 100 μL of PBS/glycerol (1:1). The solution (10 μL) was poured into the slide and observed for nuclear morphology alterations under fluorescence microscope (Olympus X 70, magnification 20 X) [43].

### Reactive oxygen species (ROS) assay

ROS production was monitored by flow cytometry using 2’,7’-dichlorodihydrofluorescin diacetate (DCFH_2_-DA). This dye is a stable non polar compound that readily diffuses into cells and is hydrolyzed by intracellular esterase to yield 2’,7’-dichloro dihydrofluorescin (DCFH), which is trapped within the cells. Hydrogen peroxide or low molecular weight peroxides produced by the cells oxidize DCFH to the highly fluorescent compound 2’,7’-dichlorofluorescein (DCF). Thus, the fluorescence intensity is proportional to the amount of hydrogen peroxide produced by the cells. Briefly, HL-60 cells (1×10^6^ cells/2 mL/well) were treated with *Z. heitzii* at different concentration for 24 h. Thirty minutes before the end of the experiment, the cell culture was treated with DCFH_2_-DA (50 μM) and kept in the dark. Cells were then collected, centrifuged (200 g; 4°C; 5 min) and the pellet was washed with 1 mL of PBS and centrifuged as mentioned earlier. The pellet was suspended in 500 μL of PBS and the fluorescence was assessed by comparing two fluorescence emission 480 nm/530 nm using a flow-cytometer (BD-LSR).

### Mitochondrial membrane potential (MMP) assay

HL-60 cells (1x10^6^ cells/2 mL/well) were treated with *Z. heitzii* extracts at different concentrations for 24 h. Thirty minutes before the end of the experiment, the cell culture was treated with Rhodamine-123 (200 nM) and kept in the dark for 30 min. Cells were then collected, centrifuged (400 g; 4°C; 5 min), the pellet was washed with 1 mL of PBS and centrifuged as mentioned earlier. The fluorescence intensity of 10,000 events was analyzed in FL-1 channel using a BD FACS Calibur (Becton Dickinson, USA) flow cytometer. The decrease in fluorescence intensity caused by mitochondrial membrane potential loss was analyzed in FL-1 channel and the change in potential membrane (Δψm) was assessed by comparing fluorescence.

### DNA content and cell cycle phase distribution

HL-60 cells (1×10^6^ cells/2 mL/well) were treated with *Z. heitzii* extracts at 20, 50, 100 μg/mL for 24 h. They were harvested and washed with 1 mL of PBS, then centrifuged 400 g for 5 min at 4°C. The pellet was suspended in 100 μL of PBS and 900 μL of hypertonic buffer (PI-25 μg/mL, RNAase-40 μg/mL, sodium citrate 0.1% and Triton-100X-0.03%) and incubated at 37°C in dark for 20 min. Finally, cells were analyzed immediately on flow cytometer FACS Calibur (Becton Dickinson, USA). The data were collected in list mode on 10,000 events and illustrated in a histogram, where the number of cells (counts) was plotted against the relative fluorescence intensity of PI (FL-2; λem: 585 nm; red fluorescence). The resulting DNA distributions were analyzed by Modfit (Verity Software House Inc., Topsham, ME) for the proportions of cells in G0-G1, S- phase, and G2-M phases of the cell cycle [44].

### Phytochemical analysis of extracts by HPLC-MS

#### Chemicals and samples

Gradient grade MeOH and acetonitrile were purchased from MERCK. Gradient grade water (18 m) was prepared by using a Purelab Option-Q elga dv25 system. All standard stock solutions (1 mg/mL) were prepared by dissolving each compound in MeOH. Standards, rosmarinic acid, trans cinnamic acid, and ferulic acid were purchased from Aldrich, caffeic acid and gallic acid from Sigma-Aldrich and all other chemicals used were obtained from Sigma. All solutions were filtered through a membrane filters (Sartorius, Ø 0.22 μm) before injection into the capillary.

#### HLPC conditions

HPLC was performed with a Shimadzu HPLC device using phenolic compound preparation techniques [45]. The detector was DAD detector SPD-M20A (max = 800 nm) while the auto sampler was an SIL–20AHT. The system controller was a CBM-20Alite, the pump was an LC-10AT and the degasser was a DGU- 20A5R. The column oven was a CTO-10ASVP and the column was GL Sciences, Inertsil ODS-3-C18 (250 × 4.60 mm) 5 μm. Mobile phases were A) 2% acetic acid, and B) methanol, and flow speed was 1.000 L/minute. Column temperature was 40°C and injection volume was 2 μL.

#### Preparation of standards

Twenty standards were used for quantitative and qualitative determination: trans-cinnamic acid [(Rt) 4.98 min], ρ-coumeric acid (Rt 3.95 min), vanillic acid (Rt 3.79 min), gallic acid (Rt 1.89 min), caffeic acid (Rt 3.72 min), ferulic acid (Rt 3.99 min)), apigenin (Rt 4.83 min), naringenin (Rt 4.85 min), luteolin (Rt 4.43 min), epicatechin (Rt 3.67 min), quercetin (Rt 4.42 min), carnosic acid (Rt. 8.55 min), chlorogenic acid (Rt 3.59 min), rosmarinic acid (Rt 3.97 min), apigenin 7-glucoside (Rt 3.89 min), oleuropein (Rt 3.969 min), amentoflavone (Rt 5.16 min), naringin (Rt 3.83 min), rutin hydrate (Rt 3.69 min), hesperidin (Rt 3.85 min). Calibration concentrations were 1, 4, 5 and 20 ppm except one, apigenin 7-glucoside, was 0.9, 1.8, 4.5, 9, and 18 ppm and injection volume were 5 μL for all standards.

#### Statistical analysis

All of the data were presented as the mean ± standard deviation (SD). The viability experiments were done in triplicates and each data point represents the average of at least 3 independent experiments. Three independent experiments were performed for other assays and one of them was chosen as results to post in this study. Statistical analyses (two group comparisons) were performed using the Student’s t-test. *p*< 0.05 was considered to be statistically significant.

## Abbreviations

MTT: 3-(4, 5-dimethylthiazole-2-yl)-2,5-diphenyltetrazolium bromide
PI: Propidium iodide
MMP: Mitochondrial mitochondrial potential
Rh-123: Rhodamine-123
DCFH_2_-DA: 2’, 7’ dichlorodihydrofluorescin diacetate
ROS: Reactive oxygen species.
